# Supplementary material for: Interaction of Arylidenechromanone/Flavanone Derivatives with Biological Macromolecules Studied as Human Serum Albumin Binding, Cytotoxic Effect, Biocompatibility Towards Red Blood Cells
Source: Molecules. 2018 Dec 1;23(12):3172. doi: 10.3390/molecules23123172 (PMC6321038; doi:10.3390/molecules23123172)
Supplement: Supplementary file 1 [file molecules-23-03172-s001.pdf]

# Supplementary materials

# Interaction of arylidenechromanone/flavanone derivatives with biological macromolecules studied as human serum albumin binding, cytotoxic effect, biocompatibility towards red blood cells

**Angelika A. Adamus-Grabicka [1], Magdalena Markowicz-Piasecka [2], Michał B. Ponczek [3], Joachim Kusz [4], Magdalena Małecka [5], Urszula Krajewska [6] and Elzbieta Budzisz [1,*]**

[1] Department of Cosmetic Raw Materials Chemistry, Faculty of Pharmacy, Medical University of Lodz, ul Muszynskiego 1, 90-151 Lodz, Poland; angelika.adamus@umed.lodz.pl; elzbieta.budzisz@umed.lodz.pl

[2] Laboratory of Bioanalysis, Department of Pharmaceutical Chemistry, Drug Analysis and Radiopharmacy, Medical University of Lodz, Muszyńskiego1, 90-151 Lodz, Poland; magdalena.markowicz@umed.lodz.pl

[3] Department of General Biochemistry, Faculty of Biology and Environmental Protection, University of Lodz, Pomorska 141/143, 90-236 Lodz, Poland; michal.ponczek@biol.uni.lodz.pl

[4] Institute of Physics, University of Silesia, Uniwersytecka 4, 40-007 Katowice, Poland; joachim.kusz@us.edu.pl

[5] Department of Physical Chemistry, Theoretical and Structural Chemistry Group, Faculty of Chemistry, University of Lodz, Pomorska 163/165, 90-236 Lodz, Poland; magdalena.malecka@chemia.uni.lodz.pl

[6] Department of Pharmaceutical Biochemistry and Molecular Diagnostics, Faculty of Pharmacy, Medical University of Lodz, Muszynskiego 1, 90-151 Lodz, Poland; urszula.krajewska@umed.lodz.pl

* Correspondence: elzbieta.budzisz@umed.lodz.pl Tel.: +42-272-55-95

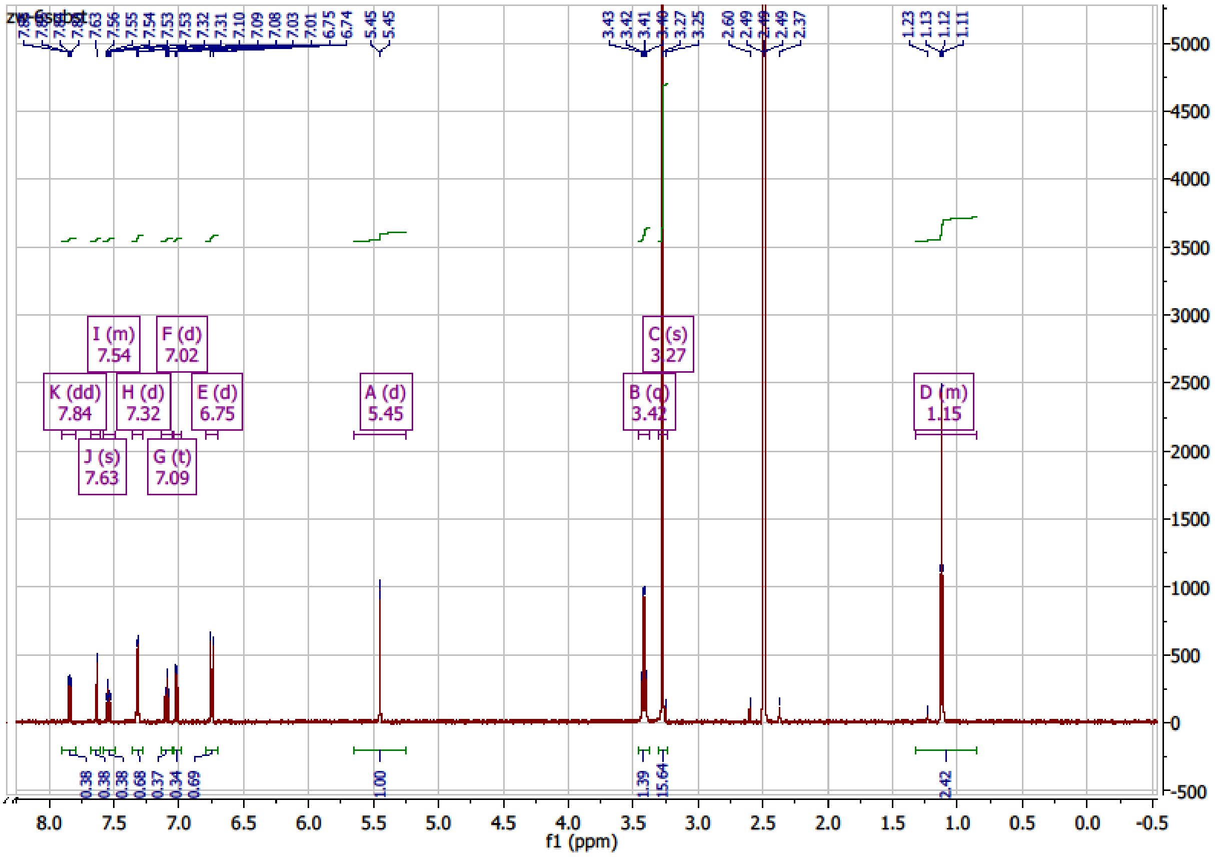


**Figure S1.** $^{1}H$ **NMR spectrum of compound 1.**

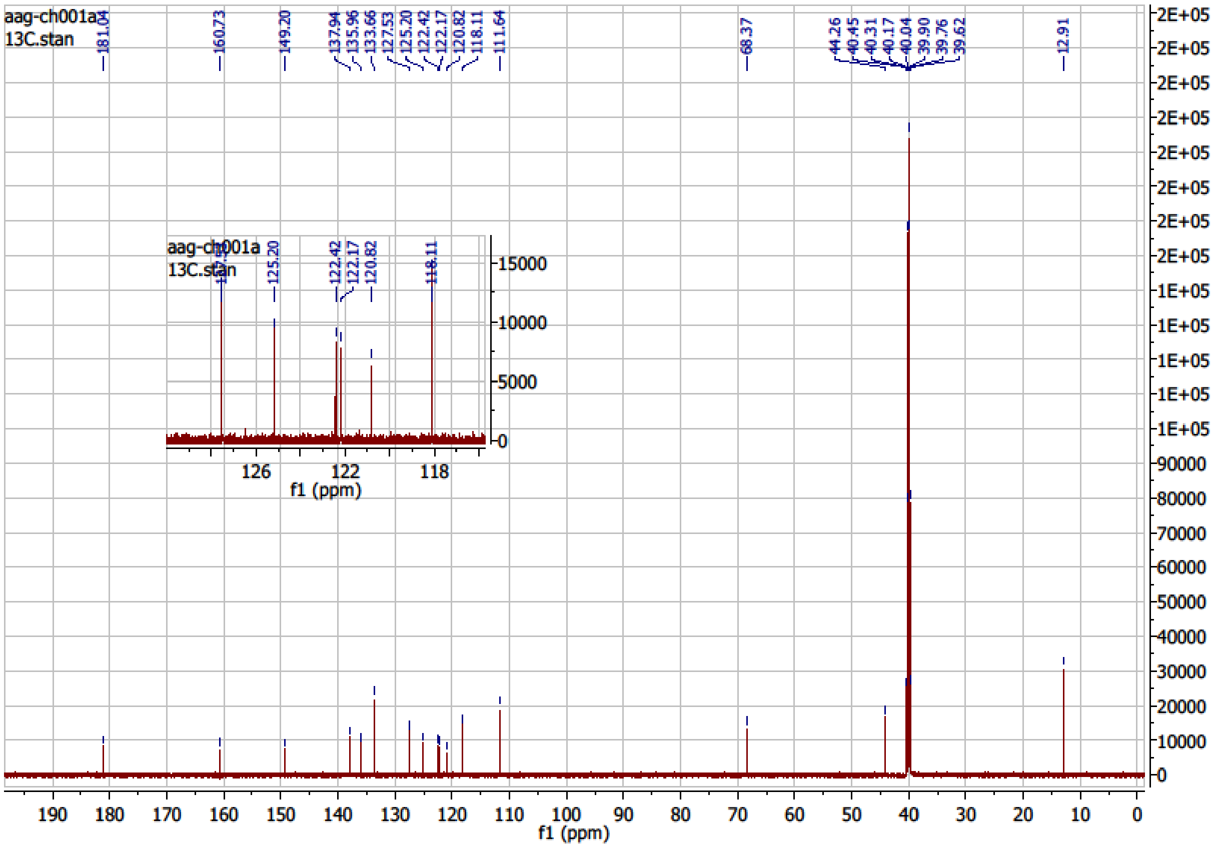


**Figure S1a.** $^{13}C$ **NMR spectrum of compound 1.**

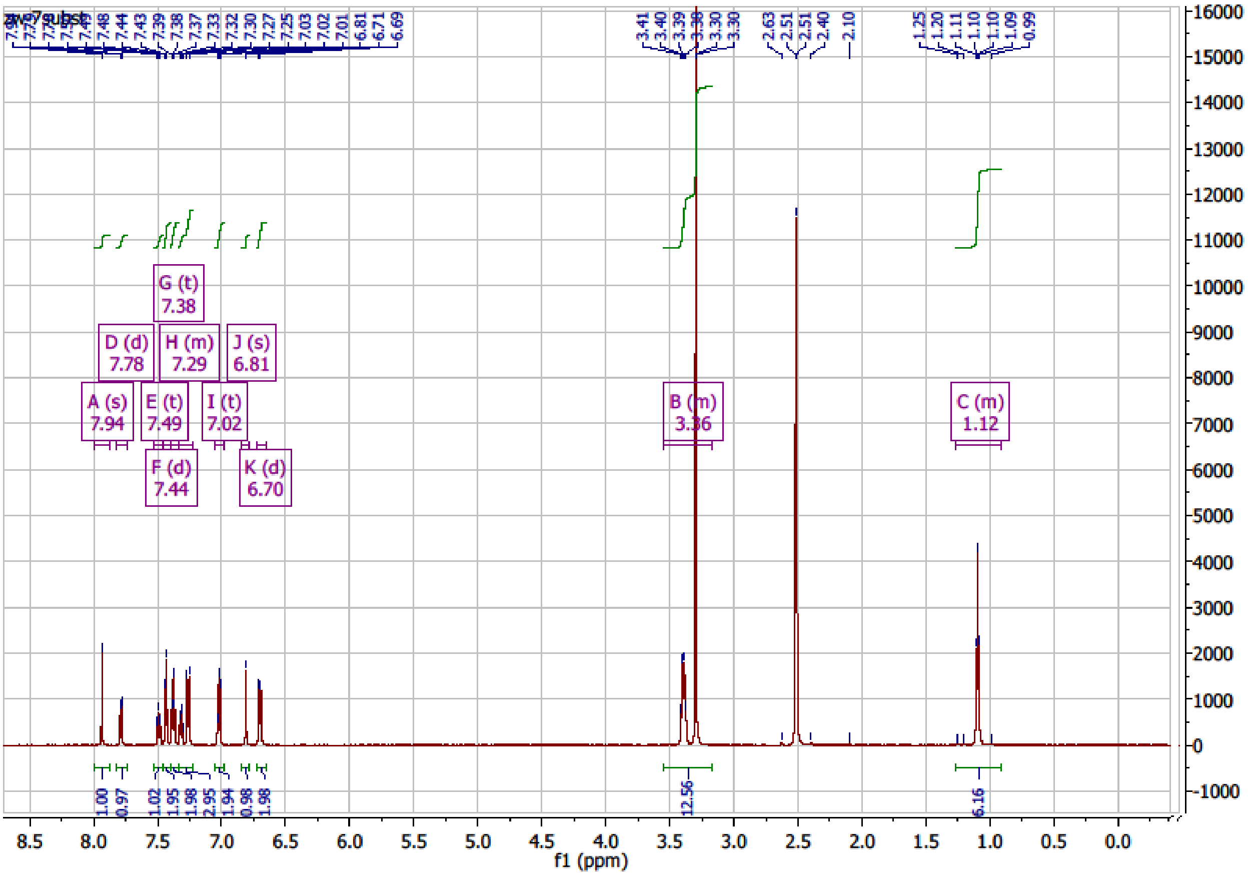


**Figure S2.** $^{1}H$ **NMR spectrum of compound 2.**

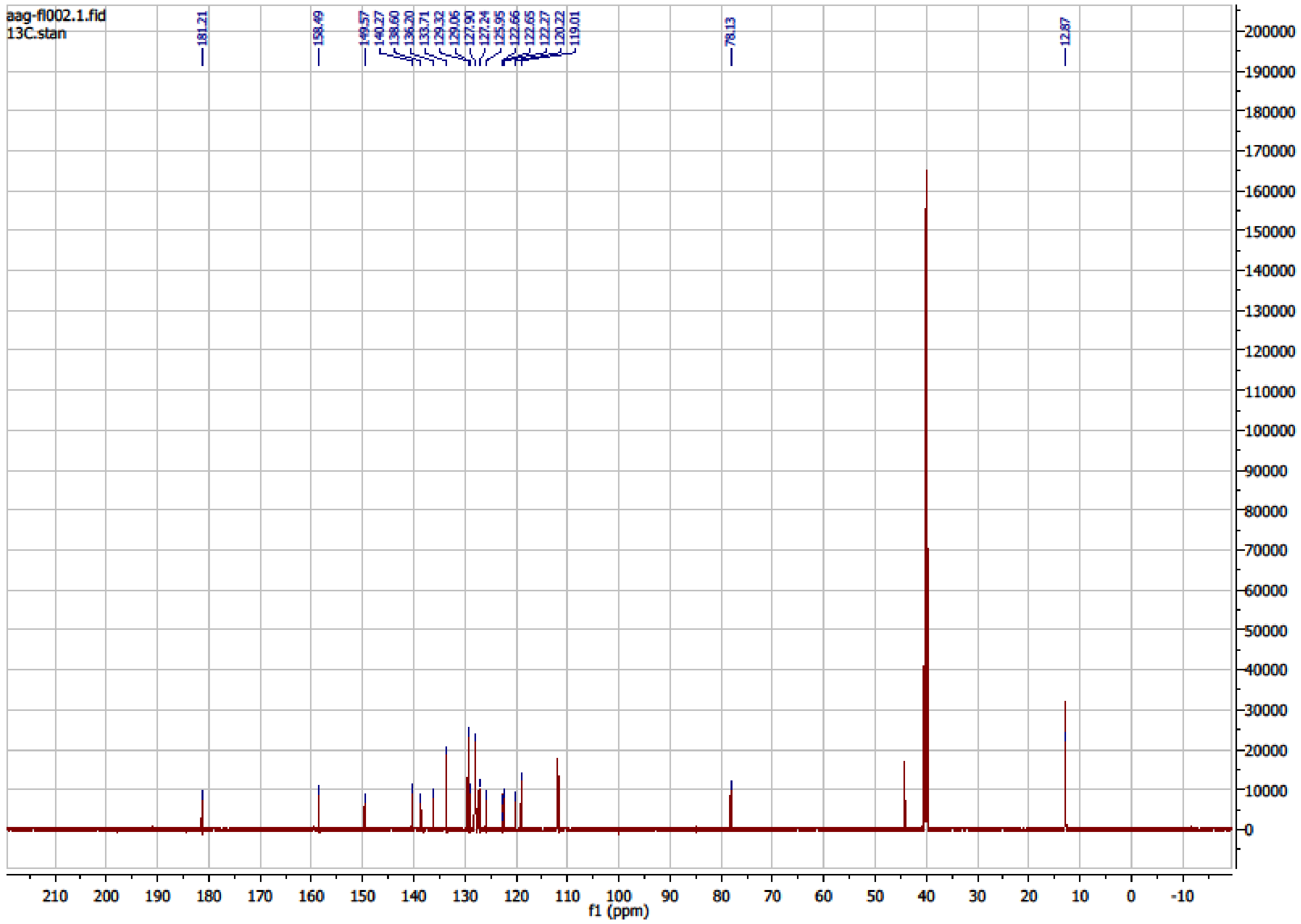


**Figure S2a.** $^{13}C$ **NMR spectrum of compound 2.**
